# The Hypnotic Screen: The Early Soviet Experiment with Film Psychotherapy

**DOI:** 10.1093/shm/hkac031

**Published:** 2022-06-21

**Authors:** Anna Toropova

## Abstract

The early Soviet period witnessed a number of experiments in ‘film psychotherapy’—the attempt to deploy the cinematic medium in hypnotherapeutic treatment. Exploring this pivotal, yet virtually unknown, moment in the history of cinema’s intertwinement with medicine, the article seeks to understand Soviet film psychotherapy as a response to transnational anxieties over cinema’s ‘powers of influence’, as well as a distinctively ‘Soviet’ experiment. An exploration of the project’s origins in Soviet psychophysiological studies of spectators and experiments in group hypnotherapeutic treatment is used to demonstrate the unique context that shaped Soviet doctors’ emergence as film therapy pioneers. After examining the medical and political hopes pinned on the project, the article tries to understand the reasons why film psychotherapy’s considerable potential remained largely unrealised. The project that promised to be a major boon to Soviet social medicine, it is argued, also brought the scientific premises of Soviet psychotherapy into question.

Free your mind from
all your worries and look at me.
You can hear music and the soft
sound of a gong. You feel well and
are calm. You are relaxing.
You do not take any notice of your
surroundings.^1^

A script for a film that employed the techniques of hypnosis to aid smoking cessation, titled *I Do Not Want to Smoke*, pictured a renowned hypnotherapist directing suggestions to an off-screen audience. As the hypnotist imparted his soothing words, a combination of rotating geometrical shapes was to appear on screen and to move in perfect time with the music and in sync with the soft beat of a gong. This mesmerising sequence of pulsating animated shapes, eye-fixation and verbal appeals to sleep was to be combined with suggestions about forgoing tobacco for the sake of the healthy mind and body required of all active ‘builders of socialism’. Published in the Soviet Union in 1936, *I Do Not Want to Smoke* (*Ia ne khochu kurit’*) was the product of a collaboration between Soviet film-makers and medical specialists. The script was written by the director Iurii Genika in consultation with Lazar’ Sukharebskii and the well-known psychotherapist Iurii Kannabikh under the auspices of the Institute of Neuro-Psychiatric Prophylaxis in Moscow. This ‘experiment in using the cinema as a direct method of therapeutic-medical treatment’, while never produced, already marked the second Soviet foray into film psychotherapy.^2^ The same team of film-makers and psychiatrists had collaborated with *Soiuzkino* to produce a film designed to facilitate the hypnotherapeutic treatment of alcoholics, entitled *Once and For All* (*Raz i navsegda*) at the beginning of the 1930s.^3^ Trials with deploying *Once and For All* in treatment were pronounced to have indicated ‘high psychotherapeutic effectiveness’.^4^

The Soviet experiment with film psychotherapy marks a pivotal, yet virtually unknown, moment in the history of cinema’s intertwinement with medicine. As Andreas Killen has shown, the therapeutic possibilities of cinema were first pictured in a number of early French feature films such as *The Mystery of the Rocks of Kador* (*Le mystère des roches de Kador*, Léonce Perret, 1912).^5^ In 1916, doctors in Germany began to discuss the possibility of films as ‘“means of treatment”’ for psychological disorders.^6^ In addition, early psychologists of film like Hugo Münsterberg had discussed the ways in which cinematic technology could be used to create a hypnotic effect.^7^ The Soviet experiment with film psychotherapy, however, marked the first attempt to turn such ambitions into reality. Bringing attention to cinema’s historical deployment in medical treatment, early Soviet efforts to exploit the psychotherapeutic capacities of film broaden our understanding of cinema’s place within medicine beyond the more well-known purposes of medical research, teaching and the dissemination of knowledge.^8^

The world’s first attempt to incorporate the medium of cinema into psychotherapeutic practice facilitates a better understanding of both the transnational history of cinema’s coincidence with political projects of human and social reform, and the ways in which cinema’s engagement with medicine has been shaped by local contexts. While film psychotherapy was one manifestation of an attempt made across Europe to harness the power of cinema to shape human minds and bodies in the early twentieth century, it was also a uniquely ‘Soviet’ experiment.^9^ Tracing the roots of film psychotherapy, considering its aims and promises, and looking closely at the techniques of the two available examples of therapeutic cinema, *I Do Not Want to Smoke* and *Once and For All*, this article unpacks Soviet film psychotherapy as a response to transnational concerns about cinema’s influence, as well as an initiative that closely reflected the unique objectives and priorities of Soviet social medicine.^10^ As I seek to show, Soviet doctors’ emphasis on group treatment and on the therapeutic benefits of hypnosis, as well as their long interest in cinema’s psycho-physiological effects, were central factors in the emergence of this ‘experiment’. Film psychotherapy’s cultural and political situatedness is nowhere more apparent than in the project’s brief lifespan. Brought to a premature end by the ideological repressions of the 1930s, the experiment that first promised to be an important boon to Soviet medicine soon became a liability. While a key means of delivering on the directives of Soviet health authorities to transform the cinema into a prime agent of ‘making the population healthy’, film hypnotherapy also threatened to bring into relief Soviet psychotherapy’s roots in the investigation of the unconscious mind.^11^

## Origins

The fate of Soviet film psychotherapy was inextricably intertwined with that of Lazar’ Sukharebskii, the medical expert and cinema theorist who was its chief proponent and set out his vision for the project in his 1936 book on the use of cinema in neurology and psychiatry, *Patho-kino*.^12^ Sukharebskii’s ideas on film psychotherapy were based on research that he had conducted at the Moscow oblast’ Scientific Research Institute of Psychoneurology and Mental Hygiene in the early 1930s.^13^ A rare example of a person whose expertise encompassed both medicine and cinema, Sukharebskii graduated from the Moscow State University’s Medical Faculty in 1922 and worked as a social hygiene propagandist.^14^ First developing an interest in scientific and educational film in the early 1920s, by the late 1930s Sukharebskii had published 12 books and over 100 articles on the use of film in health enlightenment, education and medical research.^15^ An equally prolific screenwriter, Sukharebskii headed the Department of Scientific and Educational Film at the State Institute of Cinematography (VGIK) in Moscow between 1931 and 1937.^16^ In 1936, Sukharebskii was entrusted by the Soviet Commissar for Health, Grigorii Kaminskii, to oversee the building of a film studio at Yakovenko Psychiatric Hospital in Mesherskoe selo outside Moscow. The development of film therapy was earmarked as one of the Yakovenko film studio’s main tasks, alongside producing cinematic records of patients that could be used in training and educational films.^17^

Sukharebskii envisioned film psychotherapy as a new method that could aid the collective treatment of patients in a variety of institutional settings, including psychoneurological dispensaries, health resorts, psychiatric hospitals and clinics.^18^ All existing forms of contemporary psychotherapy were thought to stand to benefit from the assistance of this new method. While Sukharebskii looked ahead to the day that film psychotherapy could fully replace hypnosis and suggestion therapy in clinics for the mentally and nervously ill, he also stressed that film psychotherapy could be just as effective in assisting work therapy, the method of ‘persuasion therapy’ pioneered by Paul Charles Dubois in Switzerland, as well as the treatment by means of emotional influence advocated by the French neurologist, Joseph Jules Dejerine.^19^ It was anticipated that film psychotherapy would be most widely deployed in the treatment of the psychoneuroses, including hysteria, phobias, mania and depression.^20^ Film psychotherapy was also thought to have a lot to offer the field of narcology, particularly the treatment of alcoholism, tobacco dependency and cocaine and morphine addiction.^21^ The moral education of young people was another area in which film psychotherapy promised to be an important resource for Soviet doctors—therapeutic cinema's capacity to help tackle unhealthy habits such as masturbation as well as sexual dysfunctions and ‘perversions’ was specifically highlighted.^22^

The product of a long-standing collaboration between Soviet psychiatrists and film-makers, the experiment with film psychotherapy grew out of a wider tradition of exchange between cinema and medicine in Soviet Russia. The main participants in film therapy already had years of experience in using cinema as a tool in medical research, training and campaigns of health enlightenment. Iurii Genika, who worked alongside Sukharebskii at VGIK, was a director of health enlightenment films such as *Alcohol* (*Alkogol*, 1927). Kannabikh had previously collaborated with Sukharebskii on the film *Neurasthenia* (*Bol’nye nervy*, Galkin, 1929), a cinematic illustration of the causes of nervous breakdown and its treatment in the Soviet Union, and on a *Soiuztekhfil’m* slide show film about the prevention of mental diseases.^23^ The lines of dialogue between psychiatry and cinema had been strengthened in the 1930s by state initiatives like the foundation of a Cinema Commission at the Soviet Commissariat of Health (Narkomzdrav). This measure sought to expand cinema’s use within medical research and teaching, and to increase the production and dissemination of sanitary enlightenment films.^24^ It was the Cinema Commission (where Sukharebskii held the post of scientific secretary) that initiated the construction of the main site allocated to the development of film psychotherapy—the Yakovenko Psychiatric Hospital film studio.^25^

The emergence of film psychotherapy reflected the heightened attentiveness of Soviet doctors, pedagogues and state officials to the mechanics of cinema’s influence on the minds and bodies of its viewers. Citing a body of Soviet and Western film theory on the unparalleled expressive capacities and emotional immediacy of the cinematic medium, Sukharebskii stressed that film therapy sought to harness the ‘tremendous psychological influence of film art on the mass audience’.^26^ Chief amongst the works that shaped Sukharebskii’s ideas about cinema’s therapeutic potential was Nikolai Lebedev’s 1935 book on cinematic specificity. Echoing the claims of early film psychologists like Hugo Münsterberg, Lebedev conceptualised the cinema as a psycho-physiological technology of unprecedented power.^27^ The medium presented a seemingly bottomless trove of visual, aural and rhythmical means of expression that not only synthesised, but surpassed the techniques of the other arts, Lebedev argued.^28^

Cinematic technology—offering a ‘new’ means of ‘objectivising human consciousness’—allowed onscreen events to unravel with the speed and dynamism that was proximate to the flow of human thought and feeling.^29^ It was not only cinema’s capacity to exteriorise the subjective experience of reality that was unique amongst the arts, Lebedev stressed, but its ability to ‘act’ on consciousness.^30^ Lebedev framed this unparalleled capacity for psychological influence as the product of cinema’s status as a ‘multi-sensory’ art form. Whereas literature acted on the senses by means of the imagination, cinema exerted a direct physiological influence on not one but two sensory organs.^31^ For Lebedev this meant that cinematic representations were endowed with a degree of ‘feeling intensity’ (*chuvstvennaia intensiv’nost’*) that was out of reach for ‘the most talented orchestra’ or the most skilled writer.^32^

Lebedev’s characterisation of cinema as a ‘multi-sensory’ art form featured centre stage in Sukharebskii account of the unprecedented ‘emotional and cognitive’ opportunities opened up by a medium that he framed as an expansive arsenal of ‘psychotherapeutic effects’.^33^ On the basis of recent research that had endowed the words of the psychotherapist with the power to impact the physiology of the patient as well as investigations into the beneficial effects of classical music on the mentally ill, Sukharebskii asserted that sound cinematography made possible the translation of a living doctor’s ‘healing words’ to a mass audience and facilitated patients’ full exposure to the therapeutic effects of music.^34^ In addition, lighting, mobile framing and editing could be used to create all manner of ‘stimuli’ that set in motion specific psychological reactions, such as mood alteration or the mobilisation of attention.^35^ Sukharebskii even looked towards using cinematic stimuli to produce bodily effects. Taking inspiration from the work of Vladimir Bekhterev and his students, Sukharebskii suggested that the rhythms created by different sound effects, music, as well as visual images and montage could be deployed in the establishment of new conditioned reflexes and the restoration of the rhythmical sensitivity often found to have diminished in the mentally ill.^36^ The rich possibilities of film psychotherapy for mental and physical ‘reforging’ were set to expand even further in the near future, Sukharebskii enthused, with advancing technological innovations such as colour cinematography and stereocinema.^37^

Sukharebskii’s conception of the film screen as a source of ‘stimuli’ that exerted a powerful effect on the viewer’s psycho-physiology was indebted to the model of enactive spectatorship—underlined by the vision of a perfect synchronicity between the viewer’s mind-body and the film screen—that Soviet film-makers and theorists like Sergei Eisenstein had articulated in the 1920s. As Ana Hedberg Olenina has shown, the assumption of a tight coordination between the spectator’s reactions and on-screen stimuli had been reinforced by psycho-physiological research on film viewers which began to be conducted in the Soviet Union from the mid-1920s.^38^ Sukharebskii, who had previously written about strategies of viewer research, directly based his experiments with film therapy on explorations of the effects of different types of films on the emotional states of healthy and mentally ill people.^39^ According to press reports, Sukharebskii’s investigations had shown that films that were cheerful in content helped to uplift patients in a depressed mood and had assisted in maintaining emotional stability. Patients suffering from nervous agitation and conditions such as epilepsy were, by contrast, found to be calmed by screenings of films featuring shots of tranquil lakes and picturesque landscapes.^40^ Sukharebskii’s research followed in the footsteps of the laboratory investigations into spectator response conducted in the Soviet Union in the late 1920s and early 1930s, which had similarly centred on the question of emotional influence. Psychologists like Abram Gel’mont had sought to investigate the effects of different ‘film stimuli’ on the emotions by recording the changes in a viewer’s pulse, breathing rate and motor control.^41^

Like many film theorists who had a background in spectator research, Sukharebskii understood cinema’s influence on the viewer’s emotions as a process of psychological contagion and unconscious mimicry.^42^ Referencing the work of the film theorists Georg Otto Stindt and Béla Balázs who had emphasised the cinema’s unparalleled gestural expressiveness and the medium’s ability to merge the spectator’s consciousness with that of the onscreen characters, Sukharebskii observed that the emotions played out on screen ‘infected’ and ‘controlled’ the audience.^43^ Sukharebskii’s assertions that ‘the viewer cries at the touching moments of a film and laughs cheerfully at humorous situations’, and that a ‘viewer who has watched a film filled with horrors feels depressed and tired afterwards’ closely paralleled the theories of emotional mimicry articulated by the first film psychologist, Hugo Münsterberg, 20 years earlier.^44^ ‘We sympathise with the sufferer and that means that the pain which he expresses becomes our own pain’, Münsterberg wrote in his 1916 work, *The Photoplay*.^45^

The central place allotted to the question of emotion in Sukharebskii’s account of film therapy reflected his conviction that cinema’s ability to shape emotional response was the lynchpin of its ‘powers of healing’. Sukharebskii was one of many Soviet doctors who stressed the power of emotion to impact physical and mental health, as well as to influence the process of recovery.^46^ Moreover, for Sukharebskii, the ‘emotional factor’ was the source of a fundamental kinship between cinema and psychotherapy. Citing doctors like Dejerine who had conceptualised the psychotherapeutic process as a means of transforming the patient’s emotional life, Sukharebskii stressed that psychotherapy, like the cinema, was primarily targeted at the sphere of feelings.^47^ The synthesis of film and psychotherapy was thereby to offer an unprecedentedly powerful means of establishing ‘healthy emotions’.^48^

## Promises

**Fig. 1:**
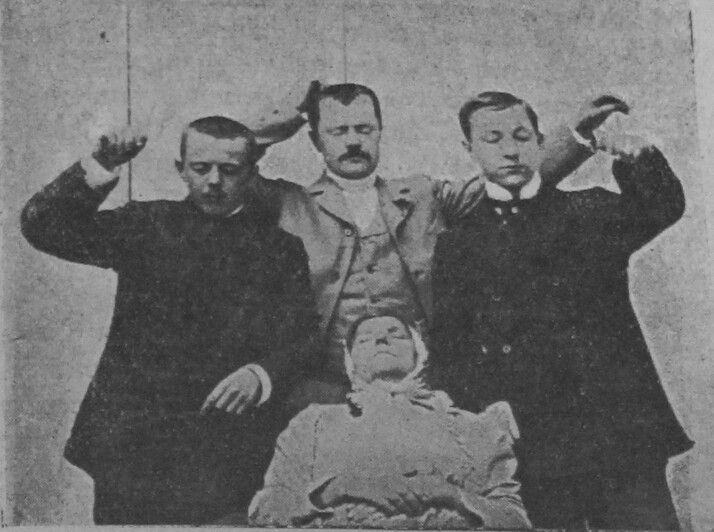
A group of alcoholics and drug addicts undergoing Vladimir Bekhterev's hypnotherapeutic treatment. *Source*: V. M Bekhterev, ‘O lechenii gipnozom’, *Vestnik znaniia*, 1926, 2, 86–96, 94.

The incorporation of cinema into therapeutic treatment promised to be an important boon to Soviet medicine. One of the most appealing features of the new method of ‘film psychotherapy’ was its facilitation of collective treatment. Film psychotherapy was to aid the form of collective psychotherapy that had been pioneered by the Russian neurologist, Vladimir Bekhterev. Having first organised a specialist outpatient clinic for the group hypnotherapeutic treatment of alcoholics in St. Petersburg alongside A. Pevnitskii in the 1900s, Bekhterev revived this method and oversaw its wide deployment at his Psychoneurological Institute in Leningrad in the early 1920s^49^ (Figure 1). Soviet medical professionals favoured collective psychotherapy over individual treatment as a method more fitting to a society in which, as the leading psychiatrist V. A. Giliarovskii noted, large numbers of people belonged to one type of collective or other.^50^ In the view of Soviet doctors, a collective form of treatment was also more adept at conveying the social significance of the patient’s recovery. In the case of smoking cessation, for example, treatment within a group was thought to encourage the patient to perceive their habit as an antisocial act as well as damaging to their individual health.^51^ While Sukharebskii stressed that psychotherapeutic films were not to be a substitute for the doctor, the type of collective treatment they facilitated certainly promised a more economic use of the doctor’s time and energy than individual psychotherapeutic treatment.^52^

Group psychotherapy was also highly valued by Soviet doctors for allowing medical treatment to be combined with health education. A typical course of psychotherapy to treat a group of smokers or alcoholics began with a health enlightenment lecture that brought patients’ attention to the detrimental effects of alcohol or tobacco use on the human body, after which the treatment turned to suggestion and hypnosis.^53^ These attempts to rationally persuade patients through sanitary enlightenment were thought to be more effectively addressed to a collective rather than to a single patient.^54^ The group setting was also designed to foster the spirit of competitiveness between patients that could help ensure the success of treatment.^55^ Patients’ ability to see others undergoing the same treatment was particularly valued by practitioners of suggestion and hypnotherapy, who noted the increase in suggestibility and the lowering of individual resistance to hypnosis that took place during group sessions.^56^

Cinema’s capacity to effectively translate the combination of persuasion, education and suggestion that formed the bedrock of Soviet group psychotherapy is amply illustrated in the two film projects that Sukharebskii was closely involved in—*Once and For All* and *I Do Not Want to Smoke*. The first parts of both films used emotive persuasion tactics in the aim of strengthening the viewer’s resolve to quit alcohol and tobacco.^57^ To impel a visceral sensation of suffocation, the opening sequence of *I Do Not Want to Smoke* layered images of cigarettes, cigars and pipes being held in the hands of men and women, young and old people, workers and educated professionals in combination shot until the screen was filled with a thick cloud of smoke. The superimposition of smokers’ hands was accompanied by music which gradually escalated in volume and tempo. Once an entanglement of hands and tobacco smoke filled the screen, the musical crescendo was to stop abruptly, preparing the viewer’s attention for the shocking image of a child’s hand holding a smoking cigarette.^58^

After capturing the viewer’s attention through stirring visuals and music, *Once and For All* and *I Do Not Want to Smoke* presented a health enlightenment lecture. A distinguished professor appeared to demonstrate the long-term effects of alcohol and tobacco consumption to an onscreen group of patients with recourse to scientific illustrations, and proceeded to detail how hypnotherapy could come to the aid of smokers and alcoholics. The ‘professor’ in *I Do Not Want to Smoke*, ‘a calming and smiling figure’ presented authoritatively in mid-shot, informed patients that nicotine (‘the poison contained in tobacco’) wreaked havoc on the cardiovascular system.^59^ Presented in the ‘style of a scientific lecture’, the information related by the professor was accompanied by detailed anatomical diagrams of the heart.^60^ After explaining the functions of the cardiovascular system, the professor warned his onscreen audience about a common smoking-related condition—atherosclerosis. The dangers of the disease were to be brought to life in a series of dissolves showcasing individuals afflicted by atherosclerosis and ‘displaying clear signs of premature aging’.^61^ Similarly, *Once and For All* unravelled the social evils of alcoholism by documenting the crippling effects of intoxication on the family, everyday life and on work performance.^62^ After persuading onscreen patients (and of course, audience members) of the imperative of smoking cessation and abstinence from alcohol, *I Do Not Want to Smoke* and *Once and For All* proceeded to illustrate the efficacy of hypnosis in helping those wishing to quit alcohol and tobacco, before finally, turning the hypnosis onto the viewing public.

## Mass Hypnosis

**Fig. 2:**
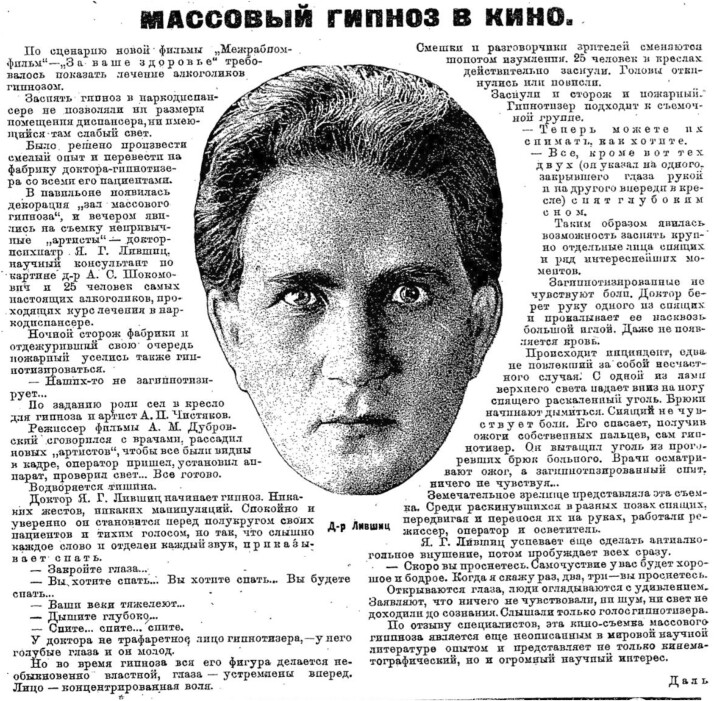
Dal’, ‘Mass Hypnosis at the Cinema’, *Sovetskii ekran*, 1928, 39,19.

By the 1920s there was an established tradition of cinematic documentions of hypnotic treatment, exemplified by the films of Max Nonne, but still no precedent for ‘mass hypnosis conducted directly from the screen’.^63^ The prospect of Soviet film-makers using the cinematic medium to impel a hypnotic trance had been first teased in 1928, in an article to promote the anti-alcoholism film, *To Your Health* (*Za vashe zdorov’e*, Aleksandr Dubrovskii, 1929). Dubrovskii’s film was still firmly placed in the tradition of cinematic representations of hypnotherapeutic treatment, making no attempt to hypnotise the viewer. Yet the title of the article publicising the film’s production in the prominent film journal *Soviet Screen*, as well as the featured film still of a hypnotist’s face gazing intently at the viewer, already conjured the prospect of ‘Mass Hypnosis in the Cinema’^64^ (Figure 2).

The lead that Soviet doctors took in incorporating film into hypnotherapeutic treatment reflected not only the priority that the Soviet medical establishment placed on harnessing the medium of cinema for the aims of public health, but the high hopes that Soviet medical professionals pinned on hypnosis. While hypnosis had also recaptured the attention of some Western specialists in the aftermath of World War I, when it rose to prominence as a treatment for shell shock and war neurosis, the medical interest in hypnosis in the Soviet Union was particularly intense and wide-ranging.^65^ Soviet medical specialists not only endorsed hypnotherapy as a treatment for neuroses and other mental disorders, but as a means of alleviating pain during childbirth and surgical operations, and as an effective means of battling addictions and unhealthy habits.^66^

The use of hypnotherapy in the treatment of alcoholics and smokers became particularly well established in the early Soviet Union. Building on a tradition of psychotherapeutic treatment developed in late Imperial Russia, eminent psychotherapists such as Vladimir Bekhterev (who had observed Jean-Martin Charcot’s hypnotic experiments at the Salpêtrière first hand in the 1890s), A. P. Nikolaev and Konstantin Platonov vouched for hypnotherapy’s high effectiveness in combatting morphinism, alcoholism and tobacco dependency.^67^ Hypnotherapy not only emerged as the dominant treatment method for neurosis and alcoholism at psychoneurological clinics in Leningrad and Kharkiv, but was also widely deployed in the new specialist narcological clinics (‘*narkodispensary*’) that opened in the 1920s.^68^

To be sure, many Soviet medical hypnotists continued to lament that the therapeutic opportunities afforded by hypnosis were not fully exploited, pointing to the legal restrictions placed on the use of hypnosis as well as public scepticism towards the method as obstacles to the widespread medical implementation of suggestion and hypnosis in the Soviet Union.^69^ Nevertheless, early Soviet doctors did make important gains in the battle to rid hypnosis of the notoriety it had gained through association with the Viennese doctor Franz Anton Mesmer.^70^ The experimental work conducted by Soviet physiologists and reflexologists played no small part in early Soviet efforts to ‘rebrand’ hypnosis as a legitimate form of medical treatment. The disciples of Bekhterev and the laboratory of Ivan Pavlov took a keen interest in hypnosis in the 1920s and sought to find a definitive physiological explanation for hypnotic phenomena.^71^ Building on Pavlov’s thesis that ‘internal inhibition and sleep’ were ‘fundamentally one and the same process’, Soviet physiologists stressed that the spread of inhibition across the brain hemispheres that took place during the normal state of sleep was also effected during hypnosis.^72^ Hypnosis was thereby unravelled as nothing other than an ordinary, sleep-like biological process which provided essential rest for the brain.^73^

Soviet film psychotherapy’s pioneering use of hypnosis to bolster the aims of public health promised to turn on its head the ‘problem’ that had increasingly perturbed medical experts and educators across Europe and the United States—cinema’s suggestive power.^74^ If early twentieth-century doctors, pedagogues and cinema reformers had worried that cinema’s powers of psychological influence were nothing short of ‘hypnotic’, *Once and For All* and *I Do Not Want to Smoke* promised to recoup cinema’s ‘suggestive power’ for the revolutionary task of psycho-physiological remaking.^75^ Much like their Western counterparts, Soviet specialists had linked commercial film consumption to physical and moral degeneration, and even criminal activity.^76^ Refracted through the lens of film psychotherapy, however, cinema’s hypnotic properties emerged as an important weapon in the battle to improve public health. The film viewer’s tendency to block out other stimuli and concentrate intensely on the screen, a tendency typically decried as dangerous by those who drew parallels between cinema and hypnosis, was recast by the pioneers of film psychotherapy as an important therapeutic opportunity. ‘[The viewer] hears the sounds emitted from the screen and switches everything else off’, Sukharebskii noted. ‘He sees the screen and perceives its images. The weighty words uttered on screen penetrate his very soul. A feeling of strength, cheerfulness and calmness grows within him. He feels better. He feels like he is stronger. He has become younger’.^77^

The attempt to lull the audience into a hypnotic sleep and to impart suggestions about smoking cessation and abstinence from alcohol comprised the third and final sections of *I Do Not Want to Smoke* and *Once and For All*. Before the hypnotisation began, however, the viewer was ‘prepared’ for what was to come through an onscreen demonstration of the professor putting a group of patients to sleep, imparting suggestions and awakening patients. The importance of explaining hypnosis to patients before commencing treatment was frequently stressed in Soviet hypnotism manuals as a means of combating the persistence of misinformation about the practice.^78^ Following the explanatory demonstration, a wide variety of techniques were called upon to impel a hypnotic state. The combination of sensory stimuli (both sudden and monotonous), and verbal suggestions used in Sukharebskii’s film projects synthesised the methods of the two predominant schools of hypnosis—the physiological method of inducing hypnosis that had been practiced by Charcot’s Parisian school and the psychological method associated with the Nancy school of Hippolyte Bernheim.^79^

Both scripts made use of the combination of intense short stimuli and weaker, more prolonged stimuli that harked back to Charcot’s hypnotic experiments at the Salpêtrière.^80^ Amongst the jolting effects outlined in the *I Do Not Want to Smoke* script were the thundering strike of a gong that initiated the hypnotisation sequence and the strobe lighting effect that accompanied the professor’s verbal countdown to 10.^81^ Estimating that approximately 20–25 per cent of patients could be successfully hypnotised by sudden loud cries of ‘sleep’, bright flashes of light, or the beating of a gong, Soviet physiologists typically explained the hypnotic effect of such unexpected, intense stimuli with reference to the state of catalepsy that animals underwent when exposed to sudden shocks.^82^ Sukharebskii’s film projects made more extensive use, however, of the type of continuous monotonous stimulation that Soviet doctors identified with being effective at hypnotising the majority of patients.^83^ During *Once and For All’*s hypnotisation sequence, a faint melody in *pianissimo* entitled ‘the theme of sleep’ was to become distinguishable out of the background orchestral score and to fleet between audibility and inaudibility.^84^ The initial loud strike of a gong in *I Do Not Want to Smoke* was to grow faint and to be repeated softly at three second intervals while the professor spoke, later being replaced by the ticking of a metronome.^85^ The efficacy of such monotonous stimuli in initiating hypnosis was typically explained by Soviet specialists with reference to Pavlov’s observation that any continuous invariable stimuli directed at a specific point of the brain eventually led to inhibition, and consequently, to sleep.^86^

Eye fixation, a hypnotisation technique staged in *Once and For All* and *I Do Not Want to Smoke* through tracking shots that slowly changed the spectator’s view of the professor from mid-shot to extreme close-up, was similarly explained by Soviet medical hypnotists as having an ‘inhibitory’ function.^87^ Fixing the patient’s gaze on a specific object or the eyes of the hypnotist, it was claimed, created a state of intense concentration that subsequently led to exhaustion, the weakening of attention, and lastly, to hypnotic sleep.^88^ The *I Do Not Want To Smoke* script included another experimental means of ‘inhibiting the activity of the higher nervous system’.^89^ As the professor addressed the viewer in the final part of the film, an animation of geometrical shapes was to appear within the shot. In strict time with the music, and in time with the sound of a gong being struck softly, these continuously moving, ‘calming’ shapes were to come in and out of focus to create a hypnotic, pulsating effect.^90^

In addition to deploying sudden and monotonous sensory stimuli, Sukharebskii’s film projects also made full use of Bernheim’s technique of sleep-inducing verbal suggestion. Targeting the arousal of ideas of sleep, both scripts pictured the professor imparting suggestions to camera that conveyed the typical sensations, thoughts and remembrances that were normally connected to sleep. *I Do Not Want to Smoke* repeatedly suggested the idea that the viewer’s senses had grown dull and that tiredness enveloped their whole body.^91^ ‘Your arms are growing heavy, your brow is also growing heavy, you want to close your eyes, your tiredness intensifies, you feel sleep encroaching’, the professor in *Once and For All* similarly suggested to the audience.^92^ The repetitive structure of the professor’s words and, most importantly, the monotonous tone in which they were to be delivered functioned as further suggestive factors.

Sukharebskii’s film projects not only exploited the unique opportunity afforded by cinema to subject the patient to a variety of stimuli simultaneously (thereby maximising the chances of successful hypnotisation), but also mobilised filmic means of concentrating audience attention.^93^ The variety of aural and visual stimuli directed at the viewer were drastically scaled back as soon as the hypnotisation process gave way to suggestions about smoking cessation and abstinence from alcohol. The musical melody and sound effects paused, the screen faded to black. The viewer was only to hear the voice of the hypnotist and to see a pair of eyes that now occupied the entire screen. Alcohol and tobacco were presented to the viewer during the course of the suggestion as poisons that stirred only visceral feelings of revulsion.^94^ ‘You find the flavour of tobacco and the smell of cigarette smoke repellent’, the professor in *I Do Not Want to Smoke* was to utter. ‘Smoking disgusts you’.^95^ The viewer’s resolve to quit unhealthy habits, and the social significance of non-smoking and abstinence, were verbally reinforced as a stark change in musical rhythm and lighting design heralded a process of awakening. ‘Remember that in battling with alcoholism’, the professor in *Once and For All* instructed, ‘you are helping not only yourself, but our great task of socialist construction, which demands from each person a clear mind, strong nerves and firm hands’. Before the screen faded to black and the sound of a rousing, uplifting march resounded, the professor noted firmly, ‘Alcoholism and socialism are incompatible.’^96^

## The Limits of the Experiment

The significant benefits that film therapy proffered to Soviet medical professionals was evidenced by the support that it found amongst the head of Narkomzdrav, Grigorii Kaminskii, as well as eminent neurologists, psychiatrists and psychotherapists such as Mikhail Krol’, Izrail’ Berger and Vladimir Zelenin.^97^ In his review of Sukharebskii’s book (titled ‘A Book of Great Contemporary Relevance’), Professor Zelenin, who headed the Clinic of Hospital Therapy at the 2^nd^ Moscow Medical Institute, asserted that its ‘original teaching on film therapy’ was being borne out by the findings of his clinic’s research into the influence of the psyche on the body.^98^ The building of the film studio at Yakovenko Psychiatric Hospital, which was to host Sukharebskii’s work on film psychotherapy, was pronounced in the film press as well as the central press as being of ‘great interest’ as the ‘world’s first attempt to harness cinema’s healing effects’.^99^ The international significance of this venture is clear from the fact that Sukharebskii’s plans to use ‘moving pictures’ ‘in the treatment of nervous and mental disorders’ and the building of the Yakovenko film studio were reported in detail in the English-language newspaper, *Moscow News*.^100^ In 1935 and 1936, the Central Institute for the Advanced Training of Physicians incorporated the question of film psychotherapy into its psychiatry course and arranged screenings of *Once and For All*.^101^ Sukharebskii was also invited to report on film psychotherapy at the Second All-Union Congress of Neuropathologists and Psychiatrists in 1936.^102^

Despite these promising beginnings, however, film psychotherapy's potential remained largely unrealised. The biggest blow to this budding project was the decision taken by the Main Directorate for Cinema (GUK) to revoke its support for Sukharebskii’s book soon after it was published. In March 1936, the *Patho-kino* book manuscript was positively reviewed by GUK’s attestation commission, which was chaired by the head of the Soviet film industry, Boris Shumiatskii. Shumiatskii approved the proposals made by VGIK to promote Sukharebskii to the title of ‘professor’ and to award him with a ‘candidate of science’ degree.^103^ Shortly after the publication of the book, however, GUK organised a public ‘denunciation’ at VGIK. According to Sukharebskii, the discussion of the book descended into a show trial, with the pre-arranged testimonies of ‘opponents’ taking centre stage and the book’s supporters being deprived of the change to speak.^104^ The criticism directed at the book at the discussion formed the pretext for Shumiatskii to remove Sukharebskii from his position at VGIK.^105^

The denunciations of Sukharebskii’s book that appeared in the Soviet press at the beginning of 1937 make clear that its swift fall from grace was closely linked to the infamous decree against the discipline of child science, or ‘pedology’. On 4 July 1936, the Party’s Central Committee issued a decree ‘On Pedological Perversions in the System of Narkompros’ which condemned pedology as a ‘pseudo-scientific’ and ‘anti-Marxist’ ‘experiment’.^106^ On 17 February 1937, a review by S. Vasil’kovskii titled ‘A Dangerous Book’ appeared in the industry newspaper *Kino*. Attacking Sukharebskii’s ‘pseudo-scientific’ work for ‘gross political errors’, the review purported that Sukharebskii not only cited pedologists but ‘propagated the ideas of pedology’ in ‘open’ defiance of the party’s resolution against the discipline.^107^ The basis for this allegation was Sukharebskii’s inclusion of films used to test children’s aptitude in his overview of the ways in which cinema had been appropriated by psychology and psychiatry.^108^ After Sukharebskii responded to these accusations, Shumiatskii personally wrote to *Kino* to reiterate the claim that Sukharebskii had attempted to revive pedology.^109^

The other criticisms levied against Sukharebskii’s book make clear that it fell victim not only to the narrowing of research parameters within Soviet science after the pedology decree, but to rising anti-internationalism, as well as the political purges that decimated the Soviet party-state at the end of the 1930s. Vasil’kovskii’s review had chastised Sukharebskii for including detailed summaries of Western films in his book and for being too dispassionate in his criticism of them. In line with the growing emphasis placed on Soviet patriotism in the late 1930s, Sukharebskii was also criticised for quoting ‘countless bourgeois (especially German scientists)’ and trumpeting their titles and achievements. Vasil’kovskii also noted that alongside including ‘meritless’ scientific works long purged from Soviet libraries, Sukharebskii’s bibliography contained ‘counter-revolutionary literature’.^110^

The call for a ban on Sukharebskii’s book by the head of the Soviet film industry immediately cast its future into doubt. The publisher, Biomedgiz, was asked by book-selling organisations whether it should be taken off the shelves and its editors were urgently called to specify whether they agreed with the published criticisms of the work.^111^ The editors of *Kino*, the newspaper where the ‘errors’ of *Patho-kino* were publicised, observed that the controversy had meant that Sukharebskii had effectively been ‘barred from working in cinema’.^112^ It was not only film psychotherapy’s chief proponent who fell out of favour in the late 1930s, but the research centre which had been central to the project’s development. The Moscow Institute of Neuro-Psychiatric Prophylaxis, where the scripts for both *Once and For All* and *I Do Not Want to Smoke* were completed, was headed by Lev Rozenshtein until 1934 and closely associated with the mental hygiene movement. Due to the discrediting of mental hygiene as a discipline following the pedology decree, the institute was transferred over to Kashchenko Psychiatric Hospital in 1938 before being restructured into the Central Institute of Psychiatry.^113^ To be sure, the pushback that GUK’s campaign against Sukharebskii received ensured that his book was not ultimately withdrawn from circulation and that he was soon able to resume work in the cinema.^114^ Indeed, only a year after the controversy, Sukharebskii was reinstated to VGIK, where the Department of Scientific and Educational Film once again voted to promote him to a professorship.^115^ Nevertheless, the section devoted to ‘film therapy’ in Sukharebskii’s 1944 book on cinema and wartime medicine made no mention of the pioneering attempt to use film to impel ‘mass hypnosis’.^116^ The topic of producing specialist films for use in psychotherapeutic treatment was not raised again until the post-Stalin period.^117^

## A Dangerous Method

While the controversy around *Patho-kino* in the wake of the 1936 pedology decree was doubtless a major factor in bringing work in the sphere of film psychotherapy to a premature end, it is striking that Sukharebskii himself expressed discomfort with crucial aspects of this ‘experiment’. Throughout his 1936 book, Sukharebskii is at pains to stress that the vast potential of film psychotherapy was by no means limited to films designed to hypnotise their audiences. ‘Straightforward’ hypnotherapeutic films in the style of *Once and For All* and *I Do Not Want to Smoke*, he asserted, presented but one configuration of the vast possible forms of psychotherapeutic film.^118^ Moreover, Sukharebskii made clear that future film projects were to swap hypnosis in favour of facilitating treatment by suggestion in the waking state.^119^ As Sukharebskii acknowledged, his vision for the future of film therapy reflected a broader trend away from ‘pure hypnotherapy’ in Soviet psychotherapy.^120^ The film specialist’s clear reservations about film hypnotherapy displayed keen awareness of the critiques that had been directed at the medical use of hypnosis.

Despite the considerable efforts that members of the Soviet scientific and medical community had made to ensure that hypnotherapy was recognised as a legitimate part of Soviet medicine by the 1930s, it nevertheless remained a contested practice. A whole host of medical specialists had voiced their distrust of a treatment method that they saw as being premised on placing the patient into an unnatural state of passivity that saw the suppression of their will and rational faculties. Doctors sounded alarm over the prospect of cultivating a ‘pathological suggestibility’ through the repeated use of hypnosis and raised concerns over the ethics of turning the patient into a ‘passive automaton’.^121^ Accordingly, dispensing with hypnosis in favour of delivering suggestions to a patient in a state of wakefulness was frequently invoked as a ‘safer’ option for the psychotherapist.^122^ Such treatment by ‘suggestion in the waking state’ was also thought closer to the form of psychotherapy that many doctors regarded to be far better suited to a socialist society—the rational persuasion of the patient through logic and argumentation. Soviet psychotherapists turned to the method of therapeutic treatment pioneered by Dubois as a means of targeting the development of the patient’s will, capacity for critical thinking, sense of ‘purposefulnes’ as well as the desire to ‘work on the self’.^123^ Since Sukharebskii’s experiments with film hypnotherapy were specifically framed as innovations in the sphere of narcology, it is particularly notable that specialists had begun to question the suitability of hypnosis for the treatment of smokers by the mid-1930s.^124^

To be sure, hypnosis remained a thriving area of medical and scientific research throughout the Stalin era. The elevation of Pavlov’s teaching to the level of doctrine after his death in 1936 ensured that his conception of hypnotic sleep as a process of brain inhibition became increasingly impervious to criticism and justified the continuation of research on the hypnotic state, as well as the deployment of hypnosis in spheres such as obstetrics, surgery and dermatology.^125^ The Pavlovian emphasis on the restorative function of sleep was also fundamental to the development of Stalin-era psychiatric treatments such as sleep therapy and insulin coma therapy.^126^ In the field of psychotherapy, however, hypnosis and suggestion largely lost ground to methods such as rational persuasion and work therapy.^127^

The conception of hypnosis as a ‘passive’ mode of treatment, while undoubtedly posing some barriers to the work of medical hypnotists in the Stalin era, was not likely to have been the determining factor in hypnotism’s fall from prominence in Soviet psychotherapy. What had become much more troublesome for Soviet psychotherapists by the 1930s—a time when specialists were obligated to clearly distinguish their methods from the ‘banned’ practice of psychoanalysis—was the tradition of scientific work that identified the hypnotic state with unconscious mental activity. From the nineteenth century, prominent medical hypnotists including Bernheim, August Forel and Albert Moll had conceived hypnosis as a means of harnessing, and better understanding, a second consciousness that existed in parallel with the normal self but was inaccessible to the waking mind.^128^ The works of both Western and Soviet doctors who had experimented with using hypnosis to explore the unconscious life of the psyche circulated widely during the early Soviet period and posed a challenge to the dominant physiological interpretation of hypnosis as a process of ‘inhibition’.^129^ If the inhibition theory of hypnosis invoked a state in which the activity of the mind was lowered or even completely paralysed (being frequently compared to the phenomenon of catalepsy in animals), the ‘alternate consciousness’ reading of hypnosis invoked a state in which the subliminal processes of the mind were fully activated.^130^

The physiological interpretation of hypnosis as a state of mental inhibition became increasingly influential during the 1920s, yet did not definitively override the rival interpretation of the hypnotic state. Indeed, even some medical hypnotists who described themselves as physiologists continued to conceive hypnosis as a state that activated unconscious thoughts and feelings. Vasilii Danilevskii, a specialist in animal hypnosis, was one such physiologist. Danilevskii proposed that the hypnotic state was premised on the substitution of conscious motivations by unconscious ones. In Danilevskii’s account, hypnosis—framed as a process in which an individual's habitual state of mind gave way to the play of the imagination, illusions and hallucinations—effected a form of ‘psychical regression’ to an earlier, more primitive form of consciousness.^131^ It is important to note that theories of the unconscious were avidly discussed in Russia and the Soviet Union until the 1930s, with many of Sigmund Freud’s works available in Russian translation and the foundation of psychoanalytic societies, institutes, clinics and schools.^132^ The attacks launched against psychoanalytic ideas and practices under Stalin, however, rendered the association of hypnosis with unconscious mental activity a source of discomfort for many psychotherapists. As Giliarovskii noted in his 1935 textbook on psychiatry, some psychotherapists had come to view hypnosis as premised on an ‘irrational factor […] linked to moments which are difficult to fully explain and which, being primarily related to the sphere of the unconscious, are perceived as something little accessible to understanding’.^133^

To be sure, Sukharebskii was careful to ally himself with the physiological interpretation of hypnosis and explained the devices used in psychotherapeutic films like *Once and For All* as having an ‘inhibitory function’.^134^ The scripts that he included in *Patho-kino*, however, painted a picture of hypnosis that more readily invoked the ‘alternate consciousness’ paradigm than the ‘inhibition’ paradigm. The hallucinatory effects created through dissolves that slowly merged shots together, superimpositions that layered images in double and triple exposure, track-ins and zoom-ins that rendered the hypnotist’s eyes the size of the entire screen, and animations that pictured floating shapes that pulsated to the rhythm of the soundtrack were difficult to reconcile with the aim of effecting a state of mental ‘switch off’. The state of consciousness targeted by these devices sooner resembled the ‘peculiar dream-consciousness’ that the doctors like Moll and Bernheim often invoked as parallels to the hypnotic state.^135^ In ‘dream-consciousness’, as in hypnosis, Moll wrote, ‘the faculty of perception is changed’, ‘we believe ourselves in another situation, and encounter all sorts of sense delusions’.^136^ Tellingly, even as physiologists framed hypnosis as a sleep-like process, they typically ignored the very question of the dream state.^137^ The process of inhibition that stood at the heart of both normal sleep and hypnosis, they stressed, impelled a state of inactivity that prevented the over-exhaustion of the brain.^138^

Sukharebskii’s film projects not only displayed a ‘dream-like’ quality but the aim of triggering the form of ‘psychical regression’ that specialists like Danilevskii had closely associated with the hypnotic state. The techniques used to present the hypnotist to the audience in *Once and For All* and *I Do Not Want to Smoke* gave striking form to Danilevskii’s thesis that the process of hypnotisation was premised on re-activating unconscious impulses—namely, unconscious feelings of fearful submission—that harked back to more ‘primitive’ phases of psychic life. ^139^ Danilevskii had contended that the technique of eye-fixation, which was usually coupled with the hypnotist’s commanding facial expression, was premised on stirring an instinctive feeling of fear before an all-powerful, predatory gaze.^140^ The psychic regression to a primal state of submission to an external force was, for Danilevskii, the primary aim of techniques that compelled the patient’s fixation on the ‘arresting’ and ‘irresistible’ eyes of the hypnotist.^141^

Danilevskii’s argument was certainly known to Iurii Kannabikh, the main psychotherapist-consultant on Sukharenskii’s film projects, who cited Danilevskii’s book in his 1929 entry on ‘Hypnotism’ for the *Great Soviet Medical Encyclopedia*.^142^ A key participant in the Soviet psychoanalytic movement who had previously written about the use of hypnosis in investigations of the unconscious mind, Kannabikh published an article in the Soviet Union’s leading psychiatry journal in 1934 where he disputed aspects of the inhibition theory of hypnosis.^143^ In line with earlier traditions of understanding hypnotic phenomena, Kannabikh continued to affirm the activation and intensification of an ‘instinctual tendency’ during the state of hypnosis, namely the ‘instinct of submission’.^144^

**Fig. 3:**
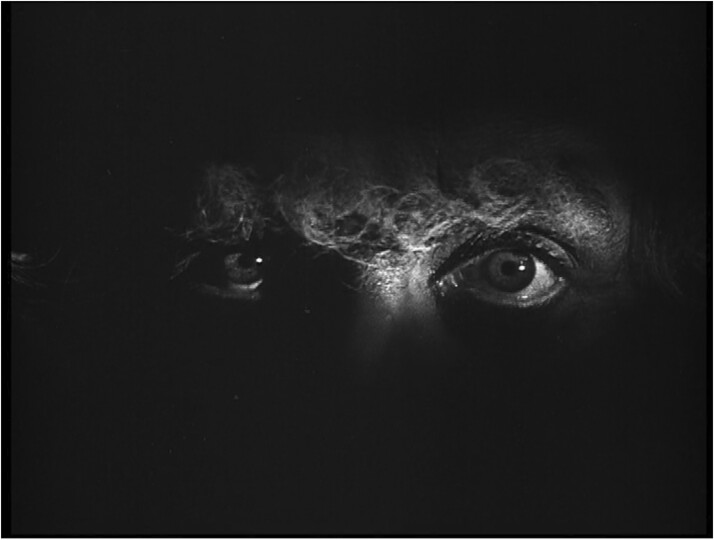
Film still from *Dr Mabuse, the Gambler* (Fritz Lang, 1922).

While the nature and extent of Kannabikh’s involvement in film psychotherapy is difficult to determine (some of Sukharebskii’s writings suggest that Kannabikh featured as the onscreen hypnotist in *Once and For All*), the film-makers’ desire to render the ‘transfixing’ power of the hypnotist’s gaze was amply demonstrated in the screenplays.^145^ The writers of *I Do Not Want to Smoke* took clear delight in using all the cinematic means available to convey the magnetising power of the hypnotist’s gaze. The professor’s final suggestions to the camera during the hypnotisation sequence, ‘You are falling asleep; You are sleeping, sleeping, sleeping’, and his measured countdown to 10 were to be recited while the camera began to slowly move forwards. The lighting during this tracking shot was to alternate between casting the professor’s face in darkness and restoring it to full visibility.^146^ The script’s further stipulation that the screen was to fade to black for 6 seconds, before the professor’s eyes, in extreme close-up, were to emerge out of the darkness, make clear the film-makers’ intentions to stir, rather than disarm, audience anxieties surrounding the figure of the hypnotist.^147^ Indeed, Sukharebskii’s commentary on the sequence betrays full awareness of its unnerving effect. ‘The face grows even larger, the forehead and chin disappear off screen. Only the bridge of the nose and the eyes remain in view. A massive pair of eyes occupies the entire screen’, he wrote.^148^ These ‘transfixing’ and ‘authoritative’ eyes were to bear down on the viewer while the professor imparted his suggestions about smoking cessation in the name of socialism. The uncanny power of the hypnotist’s gaze had been rendered in strikingly similar terms in Fritz Lang’s 1922 film *Dr Mabuse, the Gambler* (*Dr Mabuse, der Spieler*). The film’s famous hypnotisation sequence, which would have been well known to Soviet viewers, conveyed the criminal mastermind’s assertion of psychic power over his victim (the district attorney, Wenk) through an extreme close-up of Mabuse’s piercing, commanding gaze and the gradual enlargement of his face against a black background^149^ (Figure 3).

The possibility of using cinematic devices to tap into unconscious, instinctive or primitive layers of the psyche was explicitly articulated in sections of Sukharebskii’s book that dealt with the subject of rhythm. Quoting extensively from a 1926 chapter on reflexes and rhythm by one of Bekhterev’s students, G. N. Markelov, Sukharebskii endorsed his claim that rhythmical impulses ‘acted on the emotional-affective sphere and brought to life ‘paleo-psychical reactions’.^150^ The repetition of rhythmical stimuli, as well as more complex and creative processes, Sukharebskii noted, were enabled by ‘reflexive-automatic mechanisms’ that were located in the ‘subconscious sphere’ of the brain.^151^ Markelov’s theories on the innate and primal nature of the rhythmic impulse, which ‘exerted an influence on the individual from the very first day of life’ proved central to Sukharebskii’s claims on the psycho-physiological impact of the different visual and aural rhythms brought into being by psychotherapuetic films.^152^

## Sensual Thinking

Perhaps not incidentally, at the same time that work on *Once and For All* and *I Do Not Want to Smoke* was taking place, the famous Soviet director, Sergei Eisenstein, characterised rhythmical repetition and the device of the close-up as central means of activating the viewer’s regression to a pre-logical form of ‘sensual thinking’.^153^ In an essay on the ‘Art of *Mise-en-Scène*’ which was to be part of a textbook on direction that he began in 1933, Eisenstein noted that the type of rhythmical repetition used in the hypnotiser’s passes or the Voodoo cult’s ritualistic dances to bring the audience into an altered state of consciousness could be found at the basis of all aesthetic techniques of ‘bewitching’ and ‘spellbinding’ the audience.^154^ In his famous speech at the All-Union Conference of Soviet Filmworkers in 1935, Eisenstein would go on to assert that such mesmerising techniques returned the audience to an earlier stage of perception that departed from ‘the categories of practical logic’.^155^ The emotional intensity that could be achieved through the use of devices like the close-up, Eisenstein argued, was premised on its replication of ‘one of the laws of early thinking’—*pars pro toto*. The belief that ‘the part *is* simultaneously the whole’, he noted, was a pivotal example of the lack of differentiation in sensual thinking. In this diffuse, primeval form of cognition, ‘emotional and figurative constructions’ dominated and the boundaries between subject and object, the real and the imagined became blurred.^156^

Alongside the ‘rhythmic drumming’ they crafted through verbal and musical repetitions, pulsating visuals and rhythmical lighting shifts, *I Do Not Want to Smoke* and *Once and For All* harnessed a number of the other components of sensual thinking outlined by Eisenstein. In his 1935 speech, Eisenstein noted that sensual thought was devoid of the capacity for generalised abstraction, relying instead on a ‘store’ of concrete examples.^157^ The ‘decomposition’ of abstract concepts into a series of individual components was vividly demonstrated in *I Do Not Want to Smoke’*s opening sequence of smokers’ hands. The sequence began with a striking instance of *pars pro toto*; the act of smoking (the whole) was first depicted through the shot of a floating smoke ring (the part). The shot of a cigarette smoldering in a male pair of hands formed a starting point for an extensive series of superimpositions showcasing individual examples of tobacco consumption, including the image of a woman’s hands holding a slim cigarette, and the hands of an elderly man holding a pipe.^158^ The effects of smoking on the body were similarly unravelled through a multitude of concrete examples—a series of images of individual sufferers of atherosclerosis.^159^ In addition, *I Do not Want to Smoke’*s hypnotisation sequence called on the method of fragmenting the perception of an abstract concept into specific sensory impressions that Eisenstein identified in his 1935 speech with the ‘psychical gymnastics’ used by Saint Ignatius of Loyola to impel a state of religious ecstasy.^160^ In the same way as Loloya’s method compelled his followers to move from a general ‘idea’ of hell to imagine the specific sense impressions of its sounds, smells and tastes, the on-screen hypnotiser compelled the viewer to entertain ‘sleepiness’ via conjuring its specific impact on the different parts of the body and the faculties of sight and hearing.^161^

Sukharebskii’s film projects also strove to achieve the complex integration of the senses that Eisenstein conceptualised as one of the central features of the undifferentiated mode of early perception. The hypnotisation sequences in both film projects synchronised the auditory and the visual to achieve the type of unitary ‘monistic sensation’ that Eisenstein had celebrated in art forms such as the Kabuki theatre.^162^ The viewer’s exclusive concentration on the idea of sleep was encouraged in *Once and For All* through the interlacing of the hypnotists' verbal suggestions and the sound of a melody ‘on the theme of sleep’ with the visualisation of ‘narrowing consciousness’—effected via a slow track-in towards the hypnotist’s face.^163^ From its opening pairing of the auditory excess of a musical crescendo with the visual and olfactory repulsion conjured by the image of a thickening cloud of tobacco smoke, to its coordination of a rhythmical play of light and shadow with the sound of the hypnotist’s verbal countdown, instances of synaesthetic synergy similarly abounded in *I Do Not Want To Smoke*. Integrating the pulsations of geometrical shapes with the fluctuations of the musical melody and the soft, steady thumps of a gong, the script’s animated sequence fashioned a polyphony of sensory stimulation designed to reignite the viewer’s ability to ‘hear movement’ and ‘see sound’—a property, for Eisenstein, of archaic forms of perception.^164^

## Conclusion

Neither Eisenstein’s ideas about cinema opening access to an alternate sphere of consciousness, the sphere of sensual thinking, nor Sukharebskii’s attempt to impel ‘mass hypnosis’ from the screen received state support beyond the mid-1930s.^165^ The experiment with transforming the cinema’s intoxicating powers into a means of forging healthy socialist minds and bodies began to bear an uncomfortably close resemblance to the calls to harness the powers of the unconscious mind for the political task of socialist transformation that had been sounded in the 1920s.^166^ Allowing into expression a portrayal of hypnosis which departed from the dominant physiological conception of hypnotic sleep as ‘rest for the brain’, film hypnotherapy threatened the very basis of ‘scientific’ explanations of Soviet psychotherapy. The early Soviet experiment with the ‘hypnotic screen’ thereby not only presents an important testament to how cinema has helped to articulate models of subjectivity and physicality complicit with the project of biopolitical modernity, but also exemplifies cinema’s capacity to function as a technology for ‘displacing and altering’ human consciousness.^167^

## Footnotes

1 L. M. Sukharebskii, *Patokinografiia v psikhiatrii i nevropatologii* (Moscow: Biomedgiz, 1936), 162.

2
*Ibid*., 157.

3 Since *I Do Not Want to Smoke* did not go into production and *Once and For All* is lost, my analysis of these film projects will focus on the screenplays published in Sukharebskii’s 1936 book *Patho-kino* (*Patokinografiia*)*.* In 1931, the film journal *Proletarskoe kino* reported that *Once and For All*, an ‘experimental film about hypnosis’ directed by Iurii Genika was in production at the Moscow branch of *Soiuzkino*. See V. P. ‘Zvukovaia produktsiia sovetskoi kinopromyshlennosti’, *Proletarskoe kino*, 1931, 5–6, 13. There is no record of the film at *Gosfil’mofond* (the Russian State Film Archive).

4 L. M. Sukharebskii, ‘Group Psychotherapy and the Movies’, in Ralph Winn, ed, *Psychotherapy in the Soviet Union* (London: Peter Owen Ltd., 1962), 199–202, 201.

5 Andreas Killen, ‘Psychiatry and Its Visual Culture in the Modern Era’, in Greg Eghigian, ed., *The Routledge History of Madness and Mental Health* (London: Routledge, 2017), 172–90, 182–83.

6 As Killen notes, military doctors in Germany had discussed deploying the cinema as a remedy for soldiers’ ‘agitated nerves’. See his *Homo Cinematicus: Science, Motion Pictures, and the Making of Modern Germany* (Philadelphia: University of Pennsylvania Press, 2017), 44.

7 In 1916, Hugo Münsterberg had discussed the way in which the cinematic portrayal of ‘a man hypnotized in the doctor’s office’ could function to make the audience ‘become seized by the strange emotion’. See his *The Photoplay: A Psychological Study*, Allan Langdale, ed. (New York: Routledge, 2002), 108.

8 Studies of the way in which the medium of cinema has been deployed in medicine include Lisa Cartwright, *Screening the Body: Tracing Medicine’s Visual Culture* (Minneapolis: University of Minnesota Press, 1995); Christian Bonah and Anja Laukötter, ‘Moving Pictures and Medicine in the First Half of the 20th Century: Some Notes on International Historical Developments and the Potential of Medical Film Research’, *Gesnerus*, 2009, 66, 121–46; Kirsten Ostherr, *Medical Visions: Producing the Patient through Film, Television and Imaging Technologies* (New York: Oxford University Press, 2013); Scott Curtis, *The Shape of Spectatorship: Art, Science, and Early Cinema in Germany* (New York: Columbia University Press, 2015).

9 On cinema’s intertwinement with biopolitical programmes of psycho-physiological remaking, see Killen, *Homo Cinematicus;* Ute Holl, *Cinema, Trance and Cybernetics* (Amsterdam: Amsterdam University Press, 2017); Pasi Väliaho, *Mapping the Moving Image: Gesture, Thought and Cinema circa 1900* (Amsterdam: Amsterdam University Press, 2010).

10 For examples of attempts to situate Soviet social medicine within a transnational context, see Grégory Dufaud and Lara Rzesnitzek, ‘Soviet Psychiatry through the Prism of Circulation: The Case of Outpatient Psychiatry in the Interwar Period’, *Kritika*, 2016, 17.4, 781–803; Susan Gross Solomon, ‘Soviet Social Hygienists and Sexology after the Revolution: Dynamics of “Capture” at Home and Abroad’, *Ab Imperio*, 2014, 4, 107–35; David L. Hoffmann, *Cultivating the Masses: Modern State Practices and Soviet Socialism, 1914-1939* (Ithaca: Cornell University Press, 2011). Pivotal explorations of the distinguishing features of early Soviet medicine include Benjamin Zajicek, ‘Soviet Madness: Nervousness, Mild Schizophrenia, and the Professional Jurisdiction of Psychiatry in the USSR, 1918–1936’, *Ab Imperio*, 2014, 4, 167–72; Frances L. Bernstein, Christopher Burton, and Dan Healey, eds, *Soviet Medicine: Culture, Practice, and Science* (DeKalb: Northern Illinois University Press, 2010); Frances Lee Bernstein, *The Dictatorship of Sex: Lifestyle Advice for the Soviet Masses* (DeKalb, IL: Northern Illinois University Press, 2007); Kenneth Pinnow, *Lost to the Collective: Suicide and the Promise of Soviet Socialism, 1921–1929* (Ithaca: Cornell University Press, 2010). On psychotherapy in the USSR and the Eastern Bloc, see Christine Leuenberger, ‘Socialist Psychotherapy and its Dissidents’, *Journal of the History of the Behavioral Sciences*, 2001, 37.3, 261–73; Irina Sirotkina and Marina Kokorina, ‘The Dialectics of Labour in a Psychiatric Ward: Work Therapy in the Kaschenko Hospital’, in Mat Savelli and Sarah Marks, eds, *Psychiatry in Communist Europe* (Basingstoke: Palgrave Macmillan, 2015), 27–49; Sarah Marks, ‘Suggestion, Persuasion and Work: Psychotherapies in Communist Europe’, *European Journal of Psychotherapy & Counselling*, 2018, 20.1, 10–24; Aleksandra Brokman, ‘The Healing Power of Words: Psychotherapy in the USSR, 1956-1985’ (PhD dissertation, The University of East Anglia, 2018).

11 N. Semashko, ‘Kino i zdravookhranenie’, *Kino i kul’tura*, 1929, 5–6, 25–26, 26.

12 The title of Sukharebskii’s 1936 book, *Patokinografiia*, attempted to merge the words ‘pathology’ and ‘cinematography’.

13 L. M. Sukharebskii, ‘Ekran lechit’, *Vecherniaia Moskva*, 14 October 1936, 2.

14 Alexandre Sumpf, ‘Film and Anti-Alcohol Campaigns in the Soviet Union of the 1920s’, in Christian Bonah, David Cantor and Anka Laukötter, eds, *Health Education Films in the Twentieth Century* (Rochester, NY: University of Rochester Press, 2018), 198, n. 38.

15 ‘Protokol n. 17’, RGALI, f. 2900, op. 1, d. 448, ll. 20–20ob.

16
*Ibid*.

17 V. Lavrent’eva, ‘Kinostudiia psikhiatricheskoi bol’nitsy’, *Kino*, 17 October 1936, 2. The film studio’s construction was scheduled to be completed in time for the 2^nd^ Congress of Psychiatrists and Neuropathologists in December 1936.

18 Sukharebskii, ‘Ekran lechit’.

19 Sukharebskii, *Patokinografiia*, 173, 176–77, 183.

20
*Ibid*., 172.

21
*Ibid*.

22
*Ibid*.

23 Iu. V. Kannabikh and L. M. Sukharebskii, *Nervno-psikhicheskie bolezni i ikh profilaktika* (Moscow: F-ka no. 5 tresta ‘Soiuztekhfil’ma’, 1933).

24 ‘Protokol soveshchaniia po organizatsii kinokomiteta’, GARF, f. R-482, op. 25, d. 1138, ll. 1-2ob. See also I. Ierish, ‘Kinokomitet Narkomzdrava’, *Kino*, 28 September 1936, 2.

25 Lavrent’eva, ‘Kinostudiia’.

26 Sukharebskii, *Patokinografiia*, 144. Sukharebskii’s references included works by Georg Otto Stindt and Béla Balázs.

27 N. A. Lebedev, *K voprosu o spetsifike kino* (Moscow: Kinofotoizdat, 1935). ‘[T]here is surely no lack of means by which our mind can be influenced and directed in the rapid play of the pictures’, Münsterberg wrote. See his *Photoplay*, 82.

28 Lebedev, *K voprosu o spetsifike kino*, 93–9.

29
*Ibid*., 99, 96.

30
*Ibid*., 103.

31
*Ibid*., 74.

32
*Ibid*., 74, 83.

33 Sukharebskii, *Patokinografiia*, 147.

34
*Ibid*., 148–56. Konstantin Platonov’s research on the power of the spoken word as a ‘therapeutic factor’ was a key influence. See K. I. Platonov, *Slovo, kak fiziologicheskii i lechebnyi faktor* (Kharkiv: Gos. izd-vo Ukrainy, 1930). Sukharebskii also referenced the experiments with using music as means of treatment at Vladimir Bekhterev’s Psychoneurological Institute and L. S. Pavlovskaia’s research on the therapeutic effects of classical recitals on psychiatric hospital patients. See her, ‘Vliianie muzyki na dushevno-bol’nykh’, in *Sbornik posviashchennyi Vladimiru Mikhailovichu Bekhterevu k 40-letiiu professorskoi deiatel’nosti* (Leningrad: Gos. psikhonevrol. akad. i Gos. refleksol. in-t po izucheniiu mozga, 1926), 589–97.

35 Sukharebskii, *Patokinografiia*, 156.

36
*Ibid*., 166–69. Sukharebskii drew on the work of G. N. Markelov on rhythm and mental illness, see his ‘Refleksy ritma’, in *Sbornik posviashchennyi Vladimiru Mikhailovichu Bekhterevu*, 125–131.

37 Sukharebskii, *Patokinografiia*, 157.

38 Ana Hedberg Olenina, *Psychomotor Aesthetics: Movement and Affect in Modern Literature and Film* (New York: Oxford University Press, 2020), chapter 5.

39 L. M. Sukharebskii, ‘K organizatsii nauchno-issledovatel’skogo instituta sovetskoi kinematografii’, *Kino i kul’tura*, 1929, 1, 60–5.

40 ‘Kino i dushevnobol’nye’, *Izvestiia*, 11 October 1936, 4; I. O., ‘Films to Treat Mental, Nerve Disorders’, *Moscow News*, 8 October 1936, 3; Sukharebskii, ‘Ekran lechit’.

41 A. M. Gel’mont, ‘Izuchenie vliania kino na detei (problema i metody)’, *Kul’tura i kino*, 1929, 4, 38–46.

42 On Eisenstein’s conception of ‘kinesthetic empathy’ and its links to Vladimir Bekhterev’s theories of ‘emotional contagion’, see Olenina, *Psychomotor Aesthetics*, chapter 4. As Rae Beth Gordon notes, the tendency to view aesthetic experience as a process of ‘unconscious imitation’ grew out of nineteenth-century psycho-physiological aesthetic research that ‘ascribed specific emotions to certain gestures and forms’. See her *Why the French Love Jerry Lewis: From Cabaret to Early Cinema* (Stanford: Stanford University Press, 2001), 9–11.

43 Sukharebskii, *Patokinografiia*, 148. Sukharebskii was influenced by Balázs’ writings on the close-up’s capacity to convey the ‘rhythm of subjective emotions’. See his *Kul’tura kino* (Moscow: Gos. izd-vo, 1925), 39. He also referenced Balázs’ thesis that the film camera merged the viewer’s gaze and perception with those of the film’s characters. See his *Dukh fil’ma* (Moscow: Goslitizdat, 1935), 21–22. Shtindt’s writings on the expressive possibilities of the close-up, translated into Russian in 1926, were another important influence. See his *Kino-iskusstvo* (Moscow: Kinopechat’, 1926).

44 Sukharebskii, ‘Ekran lechit’.

45 Münsterberg, *The Photoplay*, 104–5. Münsterberg’s book was cited in Sukharebskii’s bibliography.

46 See, for example, G. B. Gerenshtein, *Vvedenie v prakticheskuiu psikhoterapiiu* (Leningrad: Prakticheskaia meditsina, 1927), 7–8.

47 Sukharenskii, *Patokinografiia*, 147–48. ‘[P]sychotherapy ought’, Dejerine famously asserted, ‘to address itself almost solely to the feelings, and very rarely approach the high summits of pure reason’. See his *The Psychoneuroses and their Treatment by Psychotherapy*, Smith Ely Jeliffe (trans), 2nd edn (Philadelphia: J. B. Lippincott Company, 1915), 297.

48 Sukharebskii, *Patokinografiia*, 147.

49 A. P. Nikolaev, *Teoriia i praktika gipnoza v fiziologicheskom osveshchenii* (Kyiv: Nauchnaia mysl’, 1927), 57.

50 V. A. Giliarovskii, ‘O psikhoterapii na kollektivakh nevrotikov kak osobom metode’, *Moskovskii meditsinskii zhurnal*, 1926, 7, 55–61, 57.

51 A. G. Stoiko, *Khronicheskii nikotinizm (tabakokurenie) i ego lechenie* (Gor’kii: Gor’kovskoe kraevoe izd-vo, 1935), 69.

52 Sukharebskii, *Patokinografiia*, 169–70. Specialists like P. D. Pilipenko stressed the expeditiousness and efficiency of group treatment. See his ‘Kollektivnaia psikhoterapiia’, *Sovetskaia psikhonevrologiia*, 1934, 2, 137-38.

53 N. K. Bogolepov, ‘K psikhoterapii tabakokureniia’, *Sovetskaia vrachebnaiia gazeta*, 15 May 1934, 692–93; See also Tricia Starks, ‘A Revolutionary Attack on Tobacco: Bolshevik Antismoking Campaigns in the 1920s’, *AJPH*, 2017, 107.11, 1711–17, 1716; and her chapter ‘Soviet Pioneers in Smoking Cessation: From Group Therapy in the 1920s to Cytisine in the 1970s’, in Anna Toropova and Claire Shaw, eds, *Technologies of Mind and Body in the Soviet Union and the Eastern Bloc* (London: Bloomsbury, forthcoming).

54 Stoiko, *Khronicheskii nikotinizm*, 70.

55
*Ibid*.

56 As Konstantin Platonov noted, mass hypnosis created the conditions for the laws of mimicry and psychical contagion to take effect. See K. I. Platonov and M. V. Shestopal, *Vnushenie i gipnoz v akusherstve i ginekologii* (Kharkiv: Gos. izd-vo Ukrainy, 1925), 63. See also, L. Lewenfel’d [Löwenfeld], *Gipnoz i ego tekhnika*, P. Ia. Gal’perin (trans) (Kosmos, 1927), 44, 48.

57 Soviet psychotherapists stressed the importance of ‘stirring’ the patient’s feelings through ‘affect-imbued suggestion’. See Bogolepov, ‘K psikhoterapii’, 692.

58 Sukharebskii, *Patokinografiia*, 158. The opening illustration of alcoholic intoxication in *Once and For All* was similarly designed to serve as a form of ‘emotional preparation’ (122).

59
*Ibid*., 159.

60
*Ibid*., 168.

61
*Ibid*., 160.

62
*Ibid*., 177.

63 Sukharebskii, ‘Group Psychotherapy’, 201. On Nonne’s films demonstrating his ‘cure’ of shell shocked soldiers, see Killen, *Homo Cinematicus*, 37–41.

64 Dal, ‘Massovyi gipnoz v kino’, *Sovetskii ekran*, 1928, 39, 19.

65 On the ‘upsurge in hypnotism’ after the war, see Stefan Andriopoulos, *Possessed: Hypnotic Crimes, Corporate Fiction, and the Invention of Cinema* (Chicago: University of Chicago Press, 2008), 92–93.

66 B. M. Bekhterev, ‘O lechenii gipnozom’, *Vestnik znaniia*, 1926, 2, 85–96; K. I. Platonov, *Gipnoz i vnushenie v prakticheskoi meditsine* (Kharkiv: Nauchnaia mysl’, 1925); Platonov and Shestopal, *Vnushenie i gipnoz*.

67 F. B. Tseikinskaia and N. L. Utevskii, ‘K ambulatornomu lecheniiu alkogolikov’, in *Psikhoterapiia: Sbornik statei* (Kharkiv: Gos. izd-vo, 1930), 267–83; Nikolaev, *Teoriia i praktika gipnoza*, 56; P. P. Istomin, ‘K probleme morfinizma: opyt ambulatornogo gipnosuggestivnogo lecheniia morfinistov’, in *Psikhoterapiia*, 251–65. For examples of hypnotherapy being used in the sphere of narcology in late Imperial Russia, see M. I. Bereznitskii, *Lechenie gipnozom morfinistov, alkogolikov, kuril’shchikov* (St. Petersburg: Izd. K. L. Rikkera, 1906); F. E. Rybakov, *Printsipy sovremennogo lecheniia alkogolikov* (Moscow: tipo-lit. V. Rikhter, 1905) and A. L. Mendel’son, *Psikhoterapiia alkogolizma* (St. Petersburg: ‘Tsentral’naia’ tipo-lit. M. Ia. Minkova, 1905).

68 The centers of hypnotherapeutic treatment included Bekhterev’s Psychoneurological Institute in Leningrad and the psychotherapeutic dispensary at the Ukrainian State Psychoneurological Institute. See Tseikinskaia and Utevskii, ‘K ambulatornomu lecheniiu’. On the use of hypnosis at the narcological clinics, see A. S. Sholomovich, ‘Kratkii ocherk chetyrekh let raboty’, in A. S. Sholomovich, ed, *Voprosy narkologii*, Sbornik 2 (Moscow: Moszdravotdel, 1928), 104–08; Starks, ‘A Revolutionary Attack’ and ‘Soviet Pioneers in Smoking Cessation’.

69 E. I. Dubnikov, ‘Gipnoterapiia v praktike’, *Sovetskii vrachebnyi zhurnal*, 1939, 2, 109–20. According to *Narkomzdrav* instructions, hypnosis was only permitted to be used in state medical and research institutions and could only be deployed in private clinics with special permission. A private doctor was only able to practice hypnosis if they had specialised in psychiatry and neurology for a minimum of 5 years and if another doctor was present. See Nikolaev, *Teoriia i praktika gipnoza*, 41.

70 Vladimir Rozhnov, *Gipnoz ot drevnosti do nashikh dnei* (Moscow: Sov. Rossiia, 1987), 162–84; Julia Mannherz, *Modern Occultism in Late Imperial Russia* (DeKalb, Ill.: Northern Illinois University Press, 2012), 73–4; Julia Mannherz, ‘Hypnosis in Russian Popular Culture during the Era of War and Revolution’, in Boris Kolonitskii, Steven Marks and Melissa Stockdale, eds, *Russian Culture in War and Revolution, 1914-22*, book 1 (Bloomington, Ind.: Slavica, 2014), 101–17.

71 See, for example, B. N. Birman, *Eksperimental’nyi son* (Leningrad: Gos. Izd-vo, 1925). Konstantin Platonov, Bekhterev’s student, re-assessed hypnotic phenomena from the standpoint of reflexology. See Platonov, *Slovo, kak fiziologicheskii i lechebnyi faktor*.

72 Ivan Pavlov, *Lectures on Conditioned Reflexes: Twenty-Five Years of Objective Study of the Higher Nervous Activity (Behaviour) of Animals*, Vol. 1 (London: Martin Lawrence Limited), 307. As Konstantin Platonov argued, ‘To hypnotize means to put to sleep in the most direct and ordinary sense of this word’. See Platonov and Shestopal, *Vnushenie i gipnoz*, 58.

73 Ivan Pavlov, *Lectures on Conditioned Reflexes: Conditioned Reflexes and Psychiatry*, Vol 2 (London: Lawrence and Wishart Ltd, 1941), 39–43. Boris Birman, whose experiments at Pavlov’s laboratory in the early 1920s affirmed the possibility of impelling an ‘experimental sleep’ in alert dogs, portrayed hypnosis as a naturally occurring defensive mechanism comparable to states such as animal hibernation. See Birman, *Eksperimental’nyi son*, 63.

74 On the comparison of cinema to hypnosis, see Andriopoulos, *Possessed*; Killen, *Homo Cinematicus*, ch. 3; Curtis, *Shape of Spectatorship*, 135–39; Gordon, *Why the French Love Jerry Lewis*, Chapter 5.

75 The controversy over Fritz Lang’s *The Testament of Dr. Mabuse* (1933) is a striking example of concerns about cinema’s ‘hypnotic’ influence. See Killen, *Homo Cinematicus*, 133–34.

76 Anna Toropova, ‘Science, Medicine and the Creation of a ‘Healthy’ Soviet Cinema’, *Journal of Contemporary History*, 2020, 55.1, 3–28.

77 Sukharebskii, ‘Ekran lechit’.

78 Nikolaev, *Teoriia i praktika gipnoza*, 19.

79 Ivan Pavlov, *Conditioned Reflexes: An Investigation of the Physiological Activity of the Cerebral Cortex*, G. V. Anrep (trans) (North Chelmsford, MA: Courier Corporation, 1927), 270, 405. Platonov and Shestopal, *Vnushenie i gipnoz*, 58. Bekhterev was one prominent specialist who advocated a ‘synthesis’ of the Nancy and Parisian schools. See his ‘Gipnoz, vnushenie i psikhoterapiia i ikh lechebnoe znachenie’ [1911], in *Gipnoz, vnushenie, telepatiia* (Moscow: Mysl’, 1994), 226, 241–43.

80 Bekhterev, ‘Gipnoz, vnushenie i psikhoterapiia’, 242.

81 Sukharebskii, *Patokinografiia*, shots 1 and 53, 158, 163.

82 Nikolaev, *Teoriia i praktika gipnoza*, 12.

83 Nikolaev claimed that hypnosis ensued from weak and prolonged stimuli in 75–80 per cent of cases. See *ibid*., 13.

84 Sukharebskii, *Patokinografiia*, shot 99, 180.

85
*Ibid*., shots 31 and 35, 160.

86
*Ibid*., ‘A more or less enduring stimulation falling on a certain part of the hemispheres,’ Pavlov claimed, ‘leads inevitably sooner or later to drowsiness and sleep’. See his *Lectures on Conditioned Reflexes*, 307.

87 Nikolaev, *Teoriia i praktika gipnoza*, 13–14.

88 Platonov and Shestopal, *Vnushenie i gipnoz*, 59.

89 Sukharebskii, *Patokinografiia*, 169.

90
*Ibid*., shot 52, 162.

91
*Ibid*.

92
*Ibid*., shot 102, 181. The content of these suggestions was almost identical to the examples provided in early Soviet hypnotism manuals. See Platonov and Shestopal, *Vnushenie i gipnoz*, 59–60.

93 As Nikolaev noted, most hypnotists employed different forms of stimulation simultaneously. See his *Teoriia i praktika gipnoza*, 19.

94 As Vasilii Danilevskii noted, the psychotherapist was to rouse a sense of disgust towards the unhealthy habit that the doctor sought to free the patient from. See his *Gipnotizm* (Kharkiv: Put’ prosveshcheniia, 1924), 215.

95 Sukharebskii, *Patokinografiia*, shot 54, 163.

96 Sukharebskii, *Patokinografiia*, shot 105, 182.

97 L. M. Sukharebskii, ‘Letter to the Scientific Medical Soviet of Narkomzdrav’, 25 April 1937, GARF, f. R8009, op. 2, d. 53, ll. 1–2.

98 V. Zelenin, ‘Kniga bol’shoi aktual’nosti’, GARF, f. R8009, op. 2, d. 53, 9–9ob.

99 ‘Kino i dushevnobol’nye’.

100 I. O., ‘Films to Treat Mental, Nerve Disorders’.

101 Sukharebskii, *Patokinografiia*, 110–11.

102 Sukharebskii presented twice at the congress and demonstrated a selection of psychoneurological films. See his ‘Letter to the Scientific Medical Soviet of Narkomzdrav’, 1. See also, *2-oi Vsesoiuznyi s’’ezd nevropatologov i psikhiatrov* (Moscow: 1936), 4–5.

103 M. S. Grigor’ev, A. D. Anoshchenko, A. Arsharuni, ‘Pis’mo v redaktsiiu’, *Kino*, 22 April 1937, 4.

104 Sukharebskii, ‘Letter to the Scientific Medical Soviet of Narkomzdrav’, 1; L. M. Sukharebskii, ‘Pis’mo v redaktsiiu’, *Kino*, 17 April 1937, 4.

105
*Ibid*.

106 ‘O pedologicheskikh izvrashcheniiakh v sisteme Narkomprosov’, *Pravda*, 5 July 1936, 1.

107 S. Vasil’kovskii, ‘Vrednaia kniga’, *Kino*, 17 February 1937, 3.

108 Sukharebskii, *Patokinografiia*, 66.

109 Sukharebskii, ‘Pis’mo v redaktsiiu’; B. Shumiatskii, ‘Pis’mo v redaktsiiu’, *Kino*, 22 April 1937, 4.

110 Vasil’kovskii referenced a book with a foreword by an executed ‘enemy of the people’. See his ‘Vrednaia kniga’.

111 Letter from the head of Biomedgiz to the director of the Institute of Psychoneurology and Mental Hygiene, 26 February 1937, GARF, f. R8009, op. 2, d. 53, 4.

112 ‘Ot redaktsii’, *Kino*, 17 April 1937, 4.

113 M. I. Iudin, *Ochekri istorii otechestvennoi psikhiatrii*, B. D. Petrov, ed, (Moscow: Medgiz, 1951), 389; V. N. Krasnov, ‘Moskovskii Nauchno-Issledovatel’skii Institut Psikhiatrii (k 100-letiiu so dnia osnovaniia)’, *Sotsial’naia klinicheskaia psikhiatriia*, 2020, 30.1, 5–7. On the attacks against practitioners of mental hygiene and the Institute of Neuropsychiatric Prophylaxis, see also Benjamin Zajicek, ‘Insulin Coma Therapy and the Construction of Therapeutic Effectiveness in Stalin’s Soviet Union, 1936–1953, in Savelli and Marks, eds, *Psychiatry in Communist Europe*, 53.

114 Sukharebskii’s supporters, both in the field of psychiatry and in cinematography, rallied around him. The director of the Moscow Oblast’ Scientific Institute of Psychoneurology and Mental Hygiene, I. A. Berger, dismissed the review as ‘largely having no basis in reality’. Letter from I. Berger to Biomedgiz, GARF, f. R8009, op. 2, d. 53, l. 4. The staff of VGIK also resisted the campaign, writing in to *Kino* to dispute Shumiatskii’s version of events. See Grigor’ev et al., ‘Pis’mo v redaktsiiu’.

115 From March 1938, Sukharebskii once again began to attend, and later chair, the meetings of the Department of Scientific and Educational Film at VGIK. See ‘Protokol n. 14’ and ‘Protokol n.17’, RGALI, f. 2900 (VGIK), op. 1, d. 448, ll. 13, 20ob.

116 L. M. Sukharebskii, *Voenno-sanitarnye meditsinskie i fikul’turnye kinofil’my* (Moscow: Glavkinoprokat, 1944), 34–36. This section focused solely on how feature films and documentaries could be used to lift the morale of hospital patients.

117 Sukharebskii only drew attention to this topic again in 1956, during his report at the first All-Union Conference on Questions of Psychotherapy. See his ‘Group Psychotherapy’. Some parallels might be drawn between Sukharebskii’s plans for impelling ‘mass hypnosis’ from the screen and the television ‘health séances’ led by the psychotherapist Anatolii Kashpirovsky during the *perestroika* period. On Kashpirovsky, see Simon Huxtable, ‘Remembering a Problematic Past: TV Mystics, *Perestroika* and the 1990s in Post-Soviet Media and Memory’, *European Journal of Cultural Studies*, 2017, 20.3, 307–23.

118 Sukharebskii, *Patokinografiia*, 173, 175–76.

119
*Ibid*., 146, 175–76.

120
*Ibid*., 145–46.

121 M. I. Astvatsaturov, *Psikhoterapiia i psikhoanaliz* (Petrograd: Akademicheskoe izd-vo, 1923), 8–9; Danilevskii, *Gipnotizm*, 220–21. See also V. V. Sreznevskii, *Gipnoz i vnushenie* (Petrograd: Akad. izd-vo, 1923), 51. The most virulent critique of hypnosis was made by the psychoneurologist Aron Zalkind. As he noted, the working class needed ‘the most vivid wakefulness and the most incessant criticism’ and not the ‘flight from reality’ facilitated by hypnosis. See his *Zhizn’ organizma i vnushenie* (Moscow and Leningrad: Gos. izd-vo, 1927), 125.

122 Danilevskii, *Gipnotizm*, 213.

123 Zalkind, *Zhizn’ organizma i vnushenie*, 95; Bogolepov, ‘K psikhoterapii tabakokureniia’, 694.

124 Bogolepov noted that hypnosis was not best placed to encourage the smoker to take active responsibility for the success of the treatment. See *ibid*., 693. A. G. Stoiko similarly noted that hypnotherapy could only play a modest role in smoking cessation, since it required the active participation of the patient. See his *Khronicheskii nikotinizm*, 66.

125 D. Svorad and J. Hoskovec, ‘Experimental and Clinical Study of Hypnosis in the Soviet Union and the European Socialist Countries: Bibliography’, *American Journal of Clinical Hypnosis*, 1961, 4.1, 36–46. On the ascendancy of Pavlov’s theory of sleep in the Stalin era, see Konstantin A. Bogdanov, ‘Pravo na son i uslovnye refleksy: Kolybel’nye pesni v sovetskoi kul’ture (1930–1950-e gody)’, *Novoe literaturnoe obozrenie*, 2007, 86.4, <https://magazines.gorky.media/nlo/2007/4/pravo-na-son-i-uslovnye-refleksy-kolybelnye-pesni-v-sovetskoj-kulture-1930-1950-e-gody.html>

126 Bogdanov, ‘Pravo na son’; B. Birman, ‘Primenenie terapii snom v klinike nevrozov’, *Vestnik AMN SSSR*, 1946, 5, 11–14.

127 By 1936, for example, some doctors almost exclusively advocated the use of rational persuasion in group psychotherapy. See M. G. Itkin, ‘O kollektivnom metode psikhoterapii v usloviiakh ambulatornoi obstanovki’, *Vrachebnoe delo*, 1936, 10, 885-888, Similarly, V. K. Khoroshko’s 1943 book on neuroses only recommended treatment using suggestion and hypnosis in a limited number of cases. See his *Uchenie o nevrozakh* (Moscow: Medgiz, 1943). On the widespread use of work therapy, see Sirotkina and Kokorina, ‘The Dialectics of Labour’.

128 Reassessing the common conception of a ‘rupture’ between hypnosis and psychoanalysis, Andreas Meyer has framed the emergence of hypnotism as a ‘key episode in the experimentalisation of the unconscious mind’. See his *Sites of the Unconscious: Hypnosis and the Emergence of the Psychoanalytic Setting*, Christopher Barber (trans) (Chicago: University of Chicago Press, 2013), 4. As Heather Wolffram also notes, hypnosis proved fascinating for many nineteenth-century doctors due to its facilitation of access to previously closed aspects of mental life. See her *The Stepchildren of Science: Psychical Research and Parapsychology in Germany, c. 1870-1939* (Amsterdam: Rodopi, 2009), 97. Adam Crabtree has linked the discovery of magnetic sleep to the emergence of theories of ‘alternate-consciousness’—the view of the human psyche as harbouring both a normal consciousness and a secondary, subliminal consciousness. See his *From Mesmer to Freud: Magnetic Sleep and the Roots of Psychological Healing* (New Haven, CT: Yale University Press, 1993), 289–90. See also Henri F. Ellenberger, *The Discovery of the Unconscious: The History and Evolution of Dynamic Psychiatry* (New York: Basic Books, 1970).

129 See, for example, the 1928 Russian translation of the 12th edition of Forel’s *Der Hypnotismus und die suggestive Psychotherapie* (1923)*.* On the debate over understandings of hypnotic phenomena in the early Soviet period, see Anna Toropova, ‘The Battle over Hypnosis in Early Soviet Russia’, in Toropova and Shaw, eds, *Technologies of Mind and Body.*

130 Physiologists often drew parallels between human and animal hypnosis, framing both as manifestations of ‘the reflex of passive self-protection’. See Pavlov, *Conditioned Reflexes*, 411. See also Danilevskii, *Gipnotizm*, 195–96; V.V. Sreznevskii, ‘Predislovie’, in Avgust Forel, *Gipnotizm ili vnushenie i psikhoterapiia* (Leningrad: Obrazovanie, 1928), 3–8. On the ‘alternate-consciousness’ paradigm see Crabtree, *From Mesmer to Freud*.

131 Danilevskii, *Gipnotizm*, 182, 190.

132 On the rise and fall of psychoanalysis in the Soviet Union, see Martin A. Miller, *Freud and the Bolsheviks: Psychoanalysis in Imperial Russia and the Soviet Union* (New Haven, CT: Yale University Press, 1998); Alexander Etkind, *Eros of the Impossible: The History of Psychoanalysis in Russia*, Noah and Maria Rubins (trans) (Oxford: Westview Press, 1997); Alberto Angelini, ‘History of the Unconscious in Soviet Russia: From its Origins to the Fall of the Soviet Union’, *The International Journal of Psychoanalysis*, 2008, 89.2, 369–88.

133 V. A. Giliarovskii, *Psikhiatriia: Rukovodstvo dlia vrachei i studentov*, 2nd edn (Moscow-Leningrad: Biomedgiz, 1935), 692.

134 Sukharebskii, *Patokinografiia*, 169.

135 Albert Moll, *Hypnotism* (London: Walter Scott, 1890), 232. See also, H. Bernheim, *Hypnosis and Suggestion in Psychotherapy* [1886], Christian A. Herter (trans) (New York: University Books, 1965), 141.

136 Moll, *Hypnotism*, 231, 197.

137 This omission was highlighted during the discussion after Pavlov’s lecture at the 1935 Conference of Psychiatrists, Neurologists and Neurologists. See I. Pavlov, *Dvadtsatiletnii opyt ob’’ektivnogo izucheniia vysshei nervnoi deiatel’nosti (povedeniia) zhivotnykh* (Moscow: Nauka, 1973), 560.

138 Pavlov, *Lectures on Conditioned Reflexes*, 308.

139 Danilevskii, *Gipnotizm*, 194.

140
*Ibid*., 212.

141
*Ibid*., 229–31.

142 Iu. Kannabikh, ‘Gipnotizm’, *Bol’shaiia meditsinskaia entsiklopediia*, N. A. Semashko (ed.), Vol. 7 (Moscow: Aktsionernoe obshchestvo ‘Sovetskaia Entsiklopediia’, 1929), 142–54.

143 Iu. Kannabikh, ‘K voprosu o svobody voli’, *Voprosy filosofii i psikhologii*, 1907, 87, 171–225. Kannabikh’s influential 1928 volume on the history of psychiatry had also framed psychoanalysis as a practice that had its roots in hypnosis and suggestion. See his Iu. Kannabikh, *Istoriia psikhiatrii* (Leningrad: Gos. izd-vo, 1928), 456. Kannabikh was the last president of the Russian Psychoanalytic Society. See Miller, *Freud and the Bolsheviks*, 88.

144 Iu. V. Kannabikh, ‘Mesto gipnoza v sisteme sovetskoi psikhoterapii i psikhogigieny’, *Nevropatologiia, psikhiatriia i psikhogigiena*, 1934, 3.9, 50–60, 52.

145 When describing *Once and For All* at the First All-Union Conference on Questions of Psychotherapy in 1956, Sukharebskii noted that the viewer ‘had an opportunity to see Professor Iu. V. Kannabikh putting the patients to sleep’. See his ‘Group Psychotherapy’, 201.

146 Sukharebskii, *Patokinografiia*, shot 53, 162.

147
*Ibid*., shot 54, 163.

148
*Ibid*., 155.

149 As Stefan Andriopoulos notes, the sequence was singled out by contemporary critics for subjecting the onscreen victim, as well as the audience, to the mesmerising power of Mabuse’s gaze. See his *Possessed*, 105–07. Sergei Eisenstein re-edited the film for release onto Soviet screens together with Esfir Shub. See Aleksandr Deriabin, ‘Sergei Eizhenshtein—Esfir’ Shub, “Pozolochennaia gnil’: Kino-p’esa v 6-ti chastiakh”’, *Kinovedcheskie zapiski*, 2002, 58, 144–52. I am grateful to one of the anonymous reviewers for highlighting this.

150 Markelov, ‘Refleksy ritma’, 131.

151
*Ibid*.

152
*Ibid*., 125. Sukharebskii, *Patokinografiia*, 167–68.

153 A shared interest is suggested by the fact that Kannabikh treated Eisenstein using hypnosis in the 1920s and 1930s. See Oksana Bulgakowa, *Sergei Eisenstein: A Biography* (Berlin: PotemkinPress, 1998), 79, 185. Eisenstein also observed Kannabikh’s hypnotic experiments with Aleksander Luria. See Hedberg Olenina, *Psychomotor Aesthetics*, 223. For a detailed examination of Eisenstein’s engagement with hypnosis, see Julia Vassilieva, ‘Hypnosis, Psychotechnics and the Magic of Art’, in Ian Christie and Julia Vassilieva, eds, *The Eisenstein Universe* (London: Bloomsbury, 2021), 130–151.

154 S. M. Eizenshtein, *Izbrannye proizvedeniia v 6 t*., S. I. Iutkevich (ed.), Vol. 4 (Moscow: Iskusstvo, 1966), 131. In a 1940 essay on ‘Rhythmic Drumming’, Eisenstein would elaborate on his ideas, arguing that the very technology of cinema was premised on a form of repetition that was geared towards the viewer’s ‘submission to the sphere of sensual thinking’. See his *The Primal Phenomenon: Art*, Oksana Bulgakowa (ed.), Dustin Condren (trans) (Berlin: Potemkin Press, 2017), 239.

155 S. M. Eisenstein, ‘Speeches to the All-Union Creative Conference of Soviet Filmworkers’, *Selected Works, Vol III: Writings, 1934-47*, Richard Taylor (ed.), William Powell (trans) (London: BFI, 1996), 32.

156
*Ibid*., 30.

157 S. M. Eizenshtein, *Metod*, Vol. 1, N. I. Kleiman (ed.) (Moscow: Muzei kino; Eizenshtein tsentr, 2002), 151.

158 Sukharebskii, *Patokinografiia*, shots 6–10, 158.

159
*Ibid*., shots 24–28, 160.

160 Eizenshtein, *Metod*, Vol. 1, 156.

161
*Ibid*., 157; Sukharebskii, *Patokinografiia*, shot 52, 162.

162 S. M. Eisenstein, ‘An Unexpected Juncture’ [1929], *Selected Works, Vol 1: Writings, 1922–**34,* Richard Taylor (ed. and trans) (London: BFI, 1988), 117. Eisenstein’s unpublished textbook on direction, written in 1933/34, made reference to Solomon Shereshevskii’s heightened memory capacity and synaesthesia as ‘typical’ features of ‘pre-logical’ ‘sensual thinking’. Eizenshtein, *Izbrannye proizvedeniia*, Vol. 4, 284.

163 Sukharebskii, *Patokinografiia*, shot 102, 180.

164 Eisenstein, ‘An Unexpected Juncture’, 118. As Eisenstein wrote in 1947, ‘When you have accomplished the synaesthetic integration of sound and image, you have placed the viewer’s perception under the conditions of sensual thinking, in which synaesthetic perception is the only type of perception possible—for differentiated perception does not yet exist. See his *Metod*, Vol. 1, 46.

165 For criticism of Eisenstein’s ideas about ‘prelogical thinking’, see I. Vaisfel’d, 'Teoreticheskie oshibki Eizhenshteina', in *O fil’me “Bezhin lug” S. Eizenshteina: Protiv formalizma v kinoiskusstve* (Moscow and Leningrad: Gos. izd-vo Iskusstvo, 1937), 21–41.

166 See, for example, A. B. Zalkind, ‘Freidizm i marksizm’, *Krasnaia nov’*, 1924, 4, 163–86.

167 I draw here on Pasi Väliaho’s conceptualisation of the cinema as a ‘machine for controlling and regulating’, but also ‘displacing’ human consciousness. See his ‘foreword’ in Holl, *Cinema, Trance and Cybernetics*, 10.

